# Supplementary material for: Unveiling Sri Lanka’s brain drain and labour market pressure: A study of macroeconomic factors on migration
Source: PLoS One. 2024 Mar 11;19(3):e0300343. doi: 10.1371/journal.pone.0300343 (PMC10927103; doi:10.1371/journal.pone.0300343)
Supplement: S1 Appendix — (DOCX) [file pone.0300343.s001.docx]

**S1 Appendix. Literature summary**

**Studies on the impact of GDPPCI on migration.**

| **Author(s)** | **Research topic** | **Objective(s)** | **Rational for selection of methodology** | **Time period and country** | **Conclusion** |
| --- | --- | --- | --- | --- | --- |
| F. Islam and Khan (2015) | The long run impact of immigration on labor market in an advanced economy: Evidence from US data | To examine the dynamic relationship among immigration rate, GDP per capita, and and real wage rates in the USA. | Granger causality tests, vector auto regression analysis (VAR), vector error-correction model (VECM). | 1990- 1992 - USA | The long-run causality runs from GDP per capita to migration, not vice versa. |
| Kisswani and Khan (2023) | Immigration and GDP nexus: is the association asymmetric? | The association between GDPPCI and migration has been a long-time debatable area among economists. | nonlinear autoregressive distributed lag model (NARDL), vector error correction model (VECM) | 1947-2018 – Asian countries | The long-and short-run unidirectional causality running from positive change of GDP per capita income to migration. |
| Mohamed Aslam and Alibuhtto (2023) | Workers' remittances and economic growth: new evidence from an ARDL bounds cointegration approach for Sri Lanka | To examine the long-run relationship between GDPPCI and migration in Sri Lanka. | exploratory data analysis (EDA) and inferential data analysis (IDA) tools. | 1975–2021 – Sri Lanka | EDA confirms that GDP per capita income have a positive relationship with migration. |

**Studies on the impact of unemployment on migration.**

| **Author(s)** | **Research topic** | **Objective(s)** | **Rational for selection of methodology** | **Time period and country** | **Conclusion** |
| --- | --- | --- | --- | --- | --- |
| Sevencan (2023) | Remittances, unemployment, growth and development: a panel cointegration approach | To analyses the short-run and long-run dynamics of the relationship between remittances, unemployment, economic growth and human development in developing countries. | Vector Autoregressive Model methodology (VECM), Fully Modified (FMOLS) and Dynamic Ordinary Least Squares (DOLS). | 1990- 2019 – Developing countries | The findings of this study, in low-income economies, the direction of causality is from GDP per capita income to migration in the short run. |
| Boubtane, Coulibaly, and Rault (2013) | Immigration, unemployment and GDP in the host country: Bootstrap panel Granger causality analysis on OECD countries | To examines the causality relationship between immigration, unemployment and economic growth of the host country. | Panel Granger causality testing, vector auto regression analysis (VAR) | 1980–2005 - 22 OECD countries | Only in Portugal, unemployment negatively causes migration, while in any country, immigration does not cause unemployment. |

(Table continues the next page)

| **Author(s)** | **Research topic** | **Objective(s)** | **Rational for selection of methodology** | **Time period and country** | **Conclusion** |
| --- | --- | --- | --- | --- | --- |
| Ding, Wang, Guo, & Chen, (2023) | City size, administrative rank, and Rural–Urban migration in China | To investigates the effects of city size and administrative rank on rural–urban migration in China and examines the factors that influence the city size effect. | conditional Logit models | 2016 and 2017 – China | city size and unemployment positively influence the destination of rural–urban migrants. |
| Redlin (2023) | Quod vadis? The effect of youth unemployment and demographic pressure on migration in the MENA region | To provides a quantitative assessment of migration patterns and analyses the interacting effect of the unfavourable combination of youth unemployment and demographic pressure on migration decisions. | gravity model | 1995–2020 - 19 MENA countries | youth unemployment plays a significant role in explaining emigration flows from MENA countries. |

(Table continues the next page)

| **Author(s)** | **Research topic** | **Objective(s)** | **Rational for selection of methodology** | **Time period and country** | **Conclusion** |
| --- | --- | --- | --- | --- | --- |
| Gunarathne and Jayasinghe (2021) | Factors Affecting Unemployment Duration of the Science and Arts Stream University Graduatesin Sri Lanka | To determine the factors that affect unemployment duration of graduates in order to provide the policy makers with solid foundations for their migration decision-making processes. | an ordinal logistic regression | Questioner – Sri Lanka | the Semi Parametric Cox Proportional model is appropriate to determine the relationship between graduates' time taken to obtain the first job and migration. |

**Studies on the impact of HE on migration.**

| **Author(s)** | **Research topic** | **Objective(s)** | **Rational for selection of methodology** | **Time period and country** | **Conclusion** |
| --- | --- | --- | --- | --- | --- |
| Y. Khan and Bin (2020) | Does Student Mobility Affect Trade Flows? New Evidence from Chinese Provinces | To investigates the impact and relationship of international students inflow with export and import trade | Fixed Effect (FE) and the Generalized Method of Moments (GMM) | 2000 to 2016 – China | The study proposes that the Chinese government should attach greater importance to the role of international students inflow to promote import export trade. |
| Weber and Van Mol (2023) | The student migration transition: an empirical investigation into the nexus between development and international student migration | To analyze the relationship between development and outgoing international student mobility (ISM) | vector auto regression analysis (VAR) | 2003–2018 – Developing countries | these destination country characteristics indeed have different effects for students from origin countries with different stages of development, and that these effects cannot simply be reduced to a dichotomy between developed/developing countries. |

(Table continues the next page)

| **Author(s)** | **Research topic** | **Objective(s)** | **Rational for selection of methodology** | **Time period and country** | **Conclusion** |
| --- | --- | --- | --- | --- | --- |
| Levatino (2015) | Transnational higher education and skilled migration: Evidence from Australia | To presents empirical evidence regarding the relationship between enrolment in transnational higher education (TNE) and skilled migration into the country of the institution which provides educational services. | Granger causality tests, vector auto regression analysis (VAR) | 2002–2011 – New-Zealand | More caution should be devoted to this kind of issue by developing countries when opening their education market to foreign providers. |
| Baas, 2019 | The Education-Migration Industry: International Students, Migration Policy and the Question of Skills | To focuses on student-migrants and the way they are being catered to by an emerging education-migration industry | vector auto regression analysis (VAR) | 1980–2017 – Asia-Pacific region | Here within the broader context of a burgeoning field of research which examines the growing popularity of international education across the Asia-Pacific region; the way its emergence is increasingly entangled with specific ambitions that skilled migration programmes cater to; and the way highly regulated skilled migration programmes have given rise to a migration industry in general. |

**Studies on the impact of EG on migration.**

| **Author(s)** | **Research topic** | **Objective(s)** | **Rational for selection of methodology** | **Time period and country** | **Conclusion** |
| --- | --- | --- | --- | --- | --- |
| Aneja and Praveen (2022) | International migration remittances and economic growth in Kerala: An econometric analysis | To examines the trend and pattern of international migration and the resulting inflow of remittances to Kerala | Augmented Dickey Fuller Test (ADF) for stationarity, Johansen Cointegration test followed by the vector error correction model (VECM) | 1998–2018 – India | There exists a unidirectional long-run relationship between economic growth and migration in Kerala. |
| Tipayalai (2020) | Impact of international labor migration on regional economic growth in Thailand | To examines the impact of international labor migration on regional economic growth in Thailand | Granger causality tests, vector auto regression analysis (VAR) | 2003 to 2015 - Thailand | Immigrants, and particularly high-skilled ones, have a statistically significant and positive impact on the growth of the regional economy as well as labor productivity in Thailand |
| Qutb (2022) | Migrants’ remittances and economic growth in Egypt: an empirical analysis from 1980 to 2017 | To identify the impact of economic growth on migration. | Augmented Dickey–Fuller test and Johnsen's Co-integration test, vector error correction model (VECM), Granger causality tests, vector auto regression analysis (VAR) | 1980–2017 - Egypt | Migrants’ remittances to Egypt are countercyclical in the sense that they have a long-term negative impact on economic growth |
