# Supplementary material for: Unveiling Sri Lanka’s brain drain and labour market pressure: A study of macroeconomic factors on migration
Source: PLoS One. 2024 Mar 11;19(3):e0300343. doi: 10.1371/journal.pone.0300343 (PMC10927103; doi:10.1371/journal.pone.0300343)
Supplement: S3 Appendix — (DOCX) [file pone.0300343.s003.docx]

**S2 Appendix. Stationary variable – Higher education**

| Phillips-Perron test for unit root | | | | No of observation = 35  Newly-West lags = 3 |
| --- | --- | --- | --- | --- |
| Interpolated Dickey-Fuller | | | |  |
|  | Test statistics | 1% critical value | 5% critical value | 10% critical value |
| totalgraduates_lag_1— Mackinnon approximate p- value for Z(t) = 0.0136 | | | | |
| Z(rho) | -18.114 | -17.880 | -12.820 | -10.400 |
| Z(t) | 3.330 | -3.682 | -2.972 | -2.618 |

Source: Authors’ calculation based on STATA.
