# Supplementary material for: Unveiling Sri Lanka’s brain drain and labour market pressure: A study of macroeconomic factors on migration
Source: PLoS One. 2024 Mar 11;19(3):e0300343. doi: 10.1371/journal.pone.0300343 (PMC10927103; doi:10.1371/journal.pone.0300343)
Supplement: S4 Appendix — (DOCX) [file pone.0300343.s004.docx]

**S3 Appendix. Non-stationary variables – GDPPCI, EG, Unemployment and Migration**

| Phillips-Perron test for unit root | | | | No of observation = 35  Newly-West lags = 3 |
| --- | --- | --- | --- | --- |
| Interpolated Dickey-Fuller | | | |  |
|  | Test statistics | 1% critical value | 5% critical value | 10% critical value |
| gdppercapitaln_lag_1— Mackinnon approximate p- value for Z(t) = 0.9788 | | | | |
| Z(rho) | 0.260 | -17.880 | -12.820 | -10.400 |
| Z(t) | 0.333 | -3.682 | -2.972 | -2.618 |
| Phillips-Perron test for unit root | | | | No of observation = 32  Newly-West lags = 3 |
| Interpolated Dickey-Fuller | | | |  |
|  | Test statistics | 1% critical value | 5% critical value | 10% critical value |
| totalunemployment_lag_4 — Mackinnon approximate p- value for Z(t) = 0.2493 | | | | |
| Z(rho) | -7.440 | -17.676 | -12.724 | -10.340 |
| Z(t) | -2.088 | -3.702 | -2.980 | -2.622 |
| Phillips-Perron test for unit root | | | | No of observation = 34  Newly-West lags = 3 |
| Interpolated Dickey-Fuller | | | |  |
|  | Test statistics | 1% critical value | 5% critical value | 10% critical value |
| economicgrowth_lag_2— Mackinnon approximate p- value for Z(t) = 0.0797 | | | | |
| Z(rho) | -18.432 | -17.812 | -12.788 | -10.380 |
| Z(t) | -2.668 | -3.689 | -2.975 | -2.619 |
| Phillips-Perron test for unit root | | | | No of observation = 33  Newly-West lags = 3 |
| Interpolated Dickey-Fuller | | | |  |
|  | Test statistics | 1% critical value | 5% critical value | 10% critical value |
| totalmigration_lag_3 — Mackinnon approximate p- value for Z(t) = 0.2138 | | | | |
| Z(rho) | -3.690 | -17.744 | -12.756 | -10.360 |
| Z(t) | -2.179 | -3.696 | -2.978 | -2.620 |

Source: Authors’ calculation based on STATA.
