# Supplementary material for: Unveiling Sri Lanka’s brain drain and labour market pressure: A study of macroeconomic factors on migration
Source: PLoS One. 2024 Mar 11;19(3):e0300343. doi: 10.1371/journal.pone.0300343 (PMC10927103; doi:10.1371/journal.pone.0300343)
Supplement: S5 Appendix — (DOCX) [file pone.0300343.s005.docx]

**S4 Appendix. Generated non-stationary variables into stationary variables**

| Phillips-Perron test for unit root | | | | No of observation = 34  Newly-West lags = 3 |
| --- | --- | --- | --- | --- |
| Interpolated Dickey-Fuller | | | |  |
|  | Test statistics | 1% critical value | 5% critical value | 10% critical value |
| dgdppercapitaln_lag_1— Mackinnon approximate p- value for Z(t) = 0.0018 | | | | |
| Z(rho) | -21.638 | -17.812 | -12.788 | -10.380 |
| Z(t) | -3.935 | -3.689 | -2.975 | -2.619 |
| Phillips-Perron test for unit root | | | | No of observation = 31  Newly-West lags = 3 |
| Interpolated Dickey-Fuller | | | |  |
|  | Test statistics | 1% critical value | 5% critical value | 10% critical value |
| dtotalunemployment_lag_4 — Mackinnon approximate p- value for Z(t) = 0.0000 | | | | |
| Z(rho) | -38.458 | -17.608 | -12.692 | -10.320 |
| Z(t) | -7.634 | -3.709 | -2.983 | -2.623 |
| Phillips-Perron test for unit root | | | | No of observation = 33  Newly-West lags = 3 |
| Interpolated Dickey-Fuller | | | |  |
|  | Test statistics | 1% critical value | 5% critical value | 10% critical value |
| deconomicgrowth_lag_2— Mackinnon approximate p- value for Z(t) = 0.0000 | | | | |
| Z(rho) | -42.500 | -17.744 | -12.756 | -10.360 |
| Z(t) | -7.986 | -3.696 | -2.978 | -2.620 |
| Phillips-Perron test for unit root | | | | No of observation = 32  Newly-West lags = 3 |
| Interpolated Dickey-Fuller | | | |  |
|  | Test statistics | 1% critical value | 5% critical value | 10% critical value |
| dtotalmigration_lag_3 — Mackinnon approximate p- value for Z(t) = 0.0000 | | | | |
| Z(rho) | -39.358 | -17.676 | -12.724 | -10.340 |
| Z(t) | -7.240 | -3.702 | -2.980 | -2.622 |

Source: Authors’ calculation based on STATA.
