# Supplementary material for: Unveiling Sri Lanka’s brain drain and labour market pressure: A study of macroeconomic factors on migration
Source: PLoS One. 2024 Mar 11;19(3):e0300343. doi: 10.1371/journal.pone.0300343 (PMC10927103; doi:10.1371/journal.pone.0300343)
Supplement: S6 Appendix — (DOCX) [file pone.0300343.s006.docx]

**S5 Appendix. Generated first difference for Migration**

| Phillips-Perron test for unit root | | | | No of observation = 31  Newly-West lags = 3 |
| --- | --- | --- | --- | --- |
| Interpolated Dickey-Fuller | | | |  |
|  | Test statistics | 1% critical value | 5% critical value | 10% critical value |
| dtotalmigration_lag_3 — Mackinnon approximate p- value for Z(t) = 0.0000 | | | | |
| Z(rho) | -40.645 | -17.608 | -12.692 | -10.320 |
| Z(t) | -17.124 | -3.709 | -2.983 | -2.623 |

Source: Authors’ calculation based on STATA.
