# Supplementary material for: Unveiling Sri Lanka’s brain drain and labour market pressure: A study of macroeconomic factors on migration
Source: PLoS One. 2024 Mar 11;19(3):e0300343. doi: 10.1371/journal.pone.0300343 (PMC10927103; doi:10.1371/journal.pone.0300343)
Supplement: S7 Appendix — (DOCX) [file pone.0300343.s007.docx]

**S6 Appendix. Eigenvalue stability condition summaries error and autocorrelation test**

| Eigenvalue | | | Modulus | |  | | |
| --- | --- | --- | --- | --- | --- | --- | --- |
| .738642 | | | +.3415034i | | .813767 | | |
| .738642 | | | -.3415034i | | .813767 | | |
| -.3498504 | | | +.5414933i | | .644678 | | |
| -.3498504 | | | -.5414933i | | .644678 | | |
| .3268594 | | | +.3107512i | | .451003 | | |
| .3268594 | | | -.3107512i | | .451003 | | |
| All the eigenvalue lie inside the unit circle  VAR satisfies stability condition | | | | | | | |
| Summaries error | | | | | | | |
| Variable | Observation | | Mean | Std. Dev | Min | | Max |
| Error | 32 | | .0007464 | .1524797 | -.3990564 | | .4017704 |
| Varlmar autocorrelation test | | | | | | | |
| Lag | | Chi2 | | df | | Prob > Chi2 | |
| 1 | | 39.5454 | | 25 | | 0.03246 | |
| 2 | | 30.7554 | | 25 | | 0.19725 | |
| 3 | | 30.9345 | | 25 | | 0.19120 | |
| 4 | | 15.7205 | | 25 | | 0.92290 | |
| 5 | | 30.5685 | | 25 | | 0.20371 | |
| H0: no autocorrelation at lag order | | | | | | | |

Source: Authors’ calculation based on STATA.
