# Supplementary figures and images for: Unveiling Sri Lanka’s brain drain and labour market pressure: A study of macroeconomic factors on migration
Source: PLoS One. 2024 Mar 11;19(3):e0300343. doi: 10.1371/journal.pone.0300343 (PMC10927103; doi:10.1371/journal.pone.0300343)

**
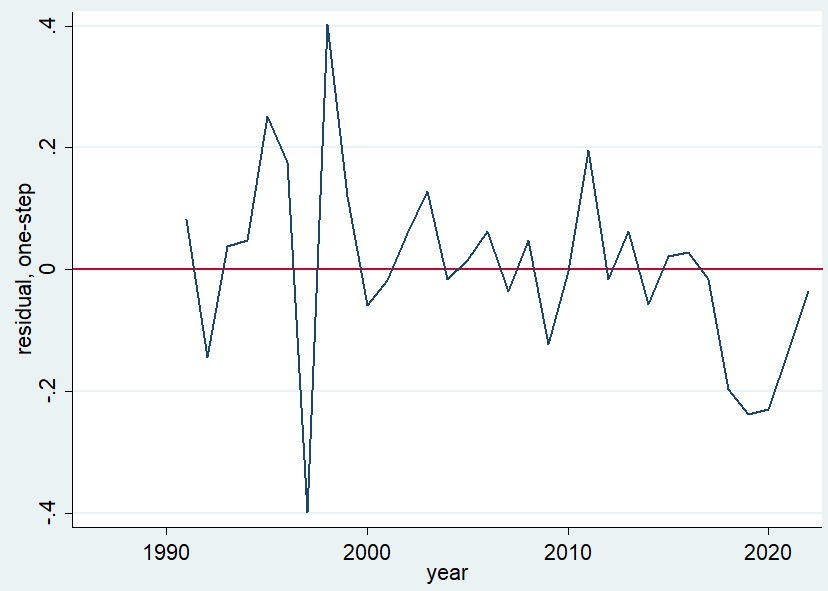
S7 Appendix. Summarized error**

Source: Authors’ demonstration based on STATA.

Supplement: S8 Appendix — (DOCX) [file pone.0300343.s008.docx]
