# Supplementary material for: Unveiling Sri Lanka’s brain drain and labour market pressure: A study of macroeconomic factors on migration
Source: PLoS One. 2024 Mar 11;19(3):e0300343. doi: 10.1371/journal.pone.0300343 (PMC10927103; doi:10.1371/journal.pone.0300343)
Supplement: S9 Appendix — (DOCX) [file pone.0300343.s009.docx]

**S8 Appendix. VAR optimum lag selection criteria**

| Selection- order criteria | | | | | | | | |
| --- | --- | --- | --- | --- | --- | --- | --- | --- |
| Sample: 1990-2022 | | |  |  |  | Numbers of obs = 33 | | |
| Lag | LL | LR | df | p | FPE | AIC | HQIC | SBIC |
| 0 | -333.12 |  |  |  | 8778.64 | 20.4315 | 20.4925 | 20.6129 |
| 1 | -235.801 | 194.64 | 16 | 0.000 | 64.1359 | 15.5031 | 15.8083 | 16.4101* |
| 2 | -210.734 | 50.134 | 16 | 0.000 | 38.8122 | 14.9536 | 15.5029 | 16.5862 |
| 3 | -198.004 | 25.461 | 16 | 0.062 | 53.5368 | 15.1518 | 15.9452 | 17.5099 |
| 4 | -167.192 | 61.624* | 16 | 0.000 | 28.1931* | 14.2541* | 15.2916* | 17.3378 |
| Endogenous: GDPPCI Unemployment HE EG | | | | | | | | |

| Selection- order criteria | | | | | | | | | | |
| --- | --- | --- | --- | --- | --- | --- | --- | --- | --- | --- |
| Sample: 1990-2022 | | |  | | |  | | Numbers of obs = 33 | | |
| Lag | LL | LR | | df | p | | FPE | AIC | HQIC | SBIC |
| 0 | -53.8704 |  | |  |  | | 1.62844 | 3.32548 | 3.34074 | 3.37083 |
| 1 | 12.571 | 132.88* | | 1 | 0.000 | | .030857* | -.640666* | -.610149* | -.549969* |
| 2 | 13.2756 | 1.4093 | | 1 | 0.235 | | .031425 | -.622765 | -.57699 | -.486719 |
| 3 | 13.6536 | .75604 | | 1 | 0.385 | | .032655 | -.585069 | -.524035 | -.403674 |
| 4 | 13.6615 | .01574 | | 1 | 0.900 | | .034719 | -.52494 | -.448648 | -.298196 |
| Endogenous: GDPPCI | | | | | | | | | | |

| Selection- order criteria | | | | | | | | | | |
| --- | --- | --- | --- | --- | --- | --- | --- | --- | --- | --- |
| Sample: 1990-2022 | | |  | | |  | | Numbers of obs = 33 | | |
| Lag | LL | LR | | df | p | | FPE | AIC | HQIC | SBIC |
| 0 | -88.9748 |  | |  |  | | 13.6694 | 5.45302 | 5.46828 | 5.49837 |
| 1 | -48.3702 | 81.209 | | 1 | 0.000 | | 1.23987 | 3.05274 | 3.08325 | 3.14343 |
| 2 | -44.7901 | 7.16 | | 1 | 0.007 | | 1.06077 | 2.89637 | 2.94215 | 3.03242 |
| 3 | -43.0975 | 3.3853 | | 1 | 0.066 | | 1.01787 | 2.85439 | 2.91543 | 3.03579 |
| 4 | -32.0717 | 22.052* | | 1 | 0.000 | | .555018* | 2.24677* | 2.32306* | 2.47352* |
| Endogenous: Unemployment | | | | | | | | | | |

| Selection- order criteria | | | | | | | | |
| --- | --- | --- | --- | --- | --- | --- | --- | --- |
| Sample: 1990-2022 | | |  |  |  | Numbers of obs = 33 | | |
| Lag | LL | LR | df | p | FPE | AIC | HQIC | SBIC |
| 0 | -134.937 |  |  |  | 221.573 | 8.23861 | 8.25387 | 8.28396 |
| 1 | -132.894 | 4.0868* | 1 | 0.043 | 208.023* | 8.17538* | 8.20589* | 8.26607* |
| 2 | -132.839 | .10921 | 1 | 0.741 | 220.368 | 8.23267 | 8.27845 | 8.36872 |
| 3 | -132.833 | .01267 | 1 | 0.910 | 234.209 | 8.29289 | 8.35393 | 8.47429 |
| 4 | -132.281 | 1.1044 | 1 | 0.293 | 240.93 | 8.32003 | 8.39633 | 8.54678 |
| Endogenous: HE | | | | | | | | |

| Selection- order criteria | | | | | | | | | |
| --- | --- | --- | --- | --- | --- | --- | --- | --- | --- |
| Sample: 1990-2022 | | | |  | |  | | Numbers of obs = 33 | |
| Lag | LL | LR | df | p | FPE | | AIC | HQIC | SBIC |
| 0 | -9.94121 |  |  |  | .113636 | | .663103 | .678362 | .708452 |
| 1 | 2.05133 | 23.985 | 1 | 0.000 | .058377 | | -.003111 | .027406 | .087586* |
| 2 | 2.05141 | .00015 | 1 | 0.990 | .062046 | | .05749 | .103266 | .193536 |
| 3 | 5.1094 | 6.116* | 1 | 0.013 | .054808* | | -.067237* | -.006203* | .114158 |
| 4 | 5.29542 | .37202 | 1 | 0.542 | .057646 | | -.017904 | .058388 | .20884 |
| Endogenous: Migration | | | | | | | | | |

| Selection- order criteria | | | | | | | | |
| --- | --- | --- | --- | --- | --- | --- | --- | --- |
| Sample: 1990-2022 | | |  |  |  | Numbers of obs = 33 | | |
| Lag | LL | LR | df | p | FPE | AIC | HQIC | SBIC |
| 0 | -85.6336 |  |  |  | 11.1636 | 5.25052 | 5.26578 | 5.29587 |
| 1 | -84.1347 | 2.9978 | 1 | 0.083 | 10.8326 | 5.22029 | 5.2508 | 5.31098 |
| 2 | -79.7447 | 8.7801* | 1 | 0.003 | 8.82381* | 5.01483* | 5.0606* | 5.15087* |
| 3 | -79.4879 | .5135 | 1 | 0.474 | 9.23678 | 5.05987 | 5.12091 | 5.24127 |
| 4 | -79.3152 | .34538 | 1 | 0.557 | 9.72293 | 5.11001 | 5.18631 | 5.33676 |
| Endogenous: EG | | | | | | | | |

Source: Authors’ calculation based on STATA.
