# Supplementary material for: Unveiling Sri Lanka’s brain drain and labour market pressure: A study of macroeconomic factors on migration
Source: PLoS One. 2024 Mar 11;19(3):e0300343. doi: 10.1371/journal.pone.0300343 (PMC10927103; doi:10.1371/journal.pone.0300343)
Supplement: S10 Appendix — (DOCX) [file pone.0300343.s010.docx]

**S9 Appendix. Diagnostic test results**

| **Variable** | **VIF** | **1/VIF** |
| --- | --- | --- |
| GDP per capita income | 2.39 | 0.418714 |
| Unemployment | 1.74 | 0.575660 |
| GDP | 1.69 | 0.590863 |
| Total graduation | 1.14 | 0.878834 |
| Mean VIF | 1.74 |  |
| Durbin-Watson test | 2.340232 | |
| Heteroscedasticity test | 0.1808 | |
| Normal distribution | 0.8510 | |

Source: Authors’ calculation based on STATA.
