# Supplementary material for: Unveiling Sri Lanka’s brain drain and labour market pressure: A study of macroeconomic factors on migration
Source: PLoS One. 2024 Mar 11;19(3):e0300343. doi: 10.1371/journal.pone.0300343 (PMC10927103; doi:10.1371/journal.pone.0300343)
Supplement: Attachment — Submitted filename: Review_Response_Plus_ONE_Labour_Mkt_Pressure_20_Dec_2023.docx [file pone.0300343.s011.docx]

The authors investigated “Unveiling Sri Lanka’s Brain Drain and Labour Market Pressure: A Study of Macroeconomic Factors on Migration” the study seeks to establish a relationship between macroeconomic factors and labour market pressure and the consequent brain drain. It is an interesting and well thought out topic, which is very important in contemporary times owing to macroeconomic distortions occasioned by poor policy formulation. I have some observations to assist the authors enhance the quality of the paper:

A. Abstract
(i) The authors said, “This study utilised Vector Auto-regression (VAR), Granger Causality Wald Tests” …. Is there any test like “Granger causality Wald test”?
(ii) The authors also indicated that they used “Multiple Regression Analysis through STATA.” … what particular regression test did they use?
(iii) The authors further indicated that “Moreover, the regression findings indicated that while GDPPCI and EG negatively impacted migration” … was it a test of impact or a test of relationship that the Regression was used for?
B. **Introduction**
(i) In the fourth paragraph of the introduction section the authors said “Young people primarily impact migration trends, exhibiting higher motivation to migrate.” … what does this mean?
C. **Literature Review**

The authors failed to give the study a theoretical foundation as they did not underpin the study with any theory. The absence of a theoretical framework is a significant omission as there are many theories that can underpin the study

D. **Data and Methodology**
(i) In the first paragraph of the data and methodology, the authors said “As GDPPCI significantly impacts a country’s poverty and income inequality, a country with a lower GDPPCI may experience increased labour migration [36]. In particular, unemployment greatly influences migration decisions, motivating individuals to seek better opportunities and recognition for their knowledge and skills, especially when they feel undervalued in their native country due to limited employment [11]. Furthermore, Weber and Van Mol [8] it demonstrated that there has been a remarkable increase in interest among …” what is this doing in the methodology section? The methodology section is supposed to focus strictly on the ‘how” of the study
(ii) Thea authors said “The regression analysis used a time-series data analysis approach” … what specific time series approach?
(iii) The authors said “Unemployment, and EG. Stationarity and stability are vital assumptions for the individual data variables and the system, supporting the validity of multivariate and bivariate modelling within the VAR model framework. All variables’ stationarity has already been established.” … how was stationarity established? The authors should explain
(iv) Apart from stationarity, time series analysis requires a cointegration test to know the long-run relationship between the variables and thus, determine the analytical technique. Why did the authors not test for cointegration?
(v) The authors failed to provide justifications for the chosen analytical techniques
E. Conclusion and Policy Recommendations
(i) In the first sentence of the conclusion section the authors said “The primary objective of this study is to analyse the impact of critical and newly identified factors on migration”? which are the critical factors? Which are the newly identified factors and how do they differ from the critical factors? Which of the statistical tests identified them as critical?
(ii) The authors further said “Therefore, the VAR model suggests a potential rejection of the null hypothesis for the unemployment, while the regression result do not provide grounds to reject the null hypothesis associated with the unemployment” what is the authors’ explanation for this conflict of outcomes for the VAR and regression model? How did the authors resolve this conflict? The authors should explain
F. **Limitations**:	The authors said first limitation is that “the VAR model highlights the significant positive influence of unemployment on migration, while the regression model reveals an insignificant one.” … How is this a limitation? I feel the authors should explain the basis of this conflict
