# Supplementary material for: Unveiling Sri Lanka’s brain drain and labour market pressure: A study of macroeconomic factors on migration
Source: PLoS One. 2024 Mar 11;19(3):e0300343. doi: 10.1371/journal.pone.0300343 (PMC10927103; doi:10.1371/journal.pone.0300343)
Supplement: Attachment — Submitted filename: Response to reviwers.docx [file pone.0300343.s013.docx]

**Point by point response to reviewers.**

Dear Editor and Reviewers,

We would like to express our gratitude to the editor and reviewers for giving us the opportunity to submit a revised draft of the manuscript. We appreciate the time and effort invested in providing feedback and suggestions. We have incorporated the suggestions made by Reviewer 1. Please note that the line numbers referred to in this document are aligned with the revised manuscript, which includes track changes.

| **Reviewers Comments** | **Comments of the Authors** |  |
| --- | --- | --- |
| 1. If the authors have adequately addressed your comments raised in a previous round of review and you feel that this manuscript is now acceptable for publication, you may indicate that here to bypass the “Comments to the Author” section, enter your conflict of interest statement in the “Confidential to Editor” section, and submit your "Accept" recommendation.   Reviewer #1: (No Response)  Reviewer #3: All comments have been addressed | Well noted your comment with thanks. |  |
| 2. Is the manuscript technically sound, and do the data support the conclusions?   The manuscript must describe a technically sound piece of scientific research with data that supports the conclusions. Experiments must have been conducted rigorously, with appropriate controls, replication, and sample sizes. The conclusions must be drawn appropriately based on the data presented.  Reviewer #1: Yes  Reviewer #3: Yes | Well noted your comment with thanks. |  |
| 3. Has the statistical analysis been performed appropriately and rigorously?   Reviewer #1: Yes  Reviewer #3: Yes | Duly noted! Thank you! |  |
| 4. Have the authors made all data underlying the findings in their manuscript fully available?   The [PLOS Data policy](http://www.plosone.org/static/policies.action#sharing) requires authors to make all data underlying the findings described in their manuscript fully available without restriction, with rare exception (please refer to the Data Availability Statement in the manuscript PDF file). The data should be provided as part of the manuscript or its supporting information, or deposited to a public repository. For example, in addition to summary statistics, the data points behind means, medians and variance measures should be available. If there are restrictions on publicly sharing data—e.g. participant privacy or use of data from a third party—those must be specified.  Reviewer #1: Yes  Reviewer #3: Yes | Noted with thanks! |  |
| 5. Is the manuscript presented in an intelligible fashion and written in standard English?   PLOS ONE does not copyedit accepted manuscripts, so the language in submitted articles must be clear, correct, and unambiguous. Any typographical or grammatical errors should be corrected at revision, so please note any specific errors here.  Reviewer #1: Yes  Reviewer #3: Yes | Thank you very much! |  |
| **REVIEWER #1** | | |
| Authors have significantly revised the paper according to previous comments. However, authors presented results including cointegration tests and VECM model results in the Data and Methodology section. It is not appropriate to explain results in the Data and Methodology section. Authors should shift these results to the Results and Discussion section. | We sincerely appreciate the reviewer’s insightful suggestion and bringing our attention regarding the cointegration test and VECM model result. In response to this valuable feedback, we have made a focused revision by excluding the cointegration test and VECM model result in the data and methodology section and shift these result to the result and discussion section in line 468-504 on page 23-25.  We hope this adjustment addresses your concern, and we remain open to further suggestions to improve the quality of the manuscript.  We are grateful for your time and constrictive feedback, which has contributed to the improve the manuscript. |  |
| **REVIEWER #3** | | |
| No Comments. To a reasonable extent, the authors have addressed the queries in my previous comments and I think the paper is good to go. | Thank you very much for the comment and we highly appreciated your time and effort contributed to this manuscript improvement. |  |
